# Bowel obstruction: a narrative review for all physicians

**DOI:** 10.1186/s13017-019-0240-7

**Published:** 2019-04-29

**Authors:** Fausto Catena, Belinda De Simone, Federico Coccolini, Salomone Di Saverio, Massimo Sartelli, Luca Ansaloni

**Affiliations:** 1 Emergency and Trauma Surgery Department, Parma University Hospital, Via Gramsci 14, 43126 Parma, Italy; 2 Emergency and Trauma Surgery Department, Cesena Hospital, Cesena, Italy; 3 General Surgery Department, Cambridge University Hospital, Cambridge, UK; 4 General Surgery Department, Macerata Hospital, Macerata, Italy

**Keywords:** Bowel obstructions, Emergency surgery, WSES guidelines, Primary care physician education

## Abstract

Small and large bowel obstructions are responsible for approximately 15% of hospital admissions for acute abdominal pain in the USA and ~ 20% of cases needing acute surgical care. Starting from the analysis of a common clinical problem, we want to guide primary care physicians in the initial management of a patient presenting with acute abdominal pain associated with intestinal obstruction.

## Backgrounds

Bowel obstruction is an important cause of morbidity and mortality accounting for nearly 30,000 deaths and more than $3 billion per year in direct medical costs; it is responsible for approximately 15% of hospital admissions for acute abdominal pain in the USA and ~ 20% of cases needing acute surgical care [1, 2].

Bowel obstruction etiology is based on a mechanical intrinsic luminal obstruction or extrinsic compression (Table 1). Adynamic ileus and colonic pseudo-obstruction are caused by a lack of enteric propulsion [3]. Colonic pseudo-obstruction and an adynamic ileus can be caused by drugs, trauma, postoperative period, metabolic disturbance, and other different basis [3, 4].

**Table 1:** Causes of bowel obstruction in adults

Small bowel obstruction cause	Percentages of cases	Large bowel obstruction cause	Percentages of cases
Adhesions	55–75	Cancer	60
Hernias	15–25	Volvulus	15–20
Malignancies	5–10	Diverticular	10
Others*	15	Others*	10

Others*: carcinomatosis, endometriosis, inflammatory bowel disease stenosis, intussusception, ischemic stenosis, radiation stenosis, postanastomotic stenosis, gallstones, foreign bodies, bezoars

In 90% of cases, small bowel obstruction is caused by adhesions, hernias, and neoplasms [5]. Adhesive small bowel obstruction represents 55–75% of small bowel obstruction cases [6] while hernias and small bowel tumors account for the remainder [2]. Large bowel obstruction is provoked by cancer in about 60% of cases [7]; volvulus and diverticular disease are responsible of other 30% [1]. Other various causes (carcinomatosis, endometriosis, inflammatory bowel disease stenosis, etc.) account for the remaining 10–15% of bowel obstructions. This review focuses on the management of bowel obstruction excluding duodenal mechanical obstruction to be better included in gastric outlet obstruction entity [8].

## Clinical case presentation

An 81-year-old woman with arterial hypertension and a laparotomic appendectomy when she was 12 years old presents to the emergency department with intermittent acute abdominal pain and vomiting. The last defecation was 2 days ago, and the bowel is closed to gas. Current medications only include valsartan 80 mg daily. The body temperature is 37.5 °C, and all vital parameters are normal. The remainder of the examination demonstrates pain and localized peritonism in the lower right quadrant. Laboratory tests are normal except white blood cells at 14,000 per microliter.

**Fig. 1:**
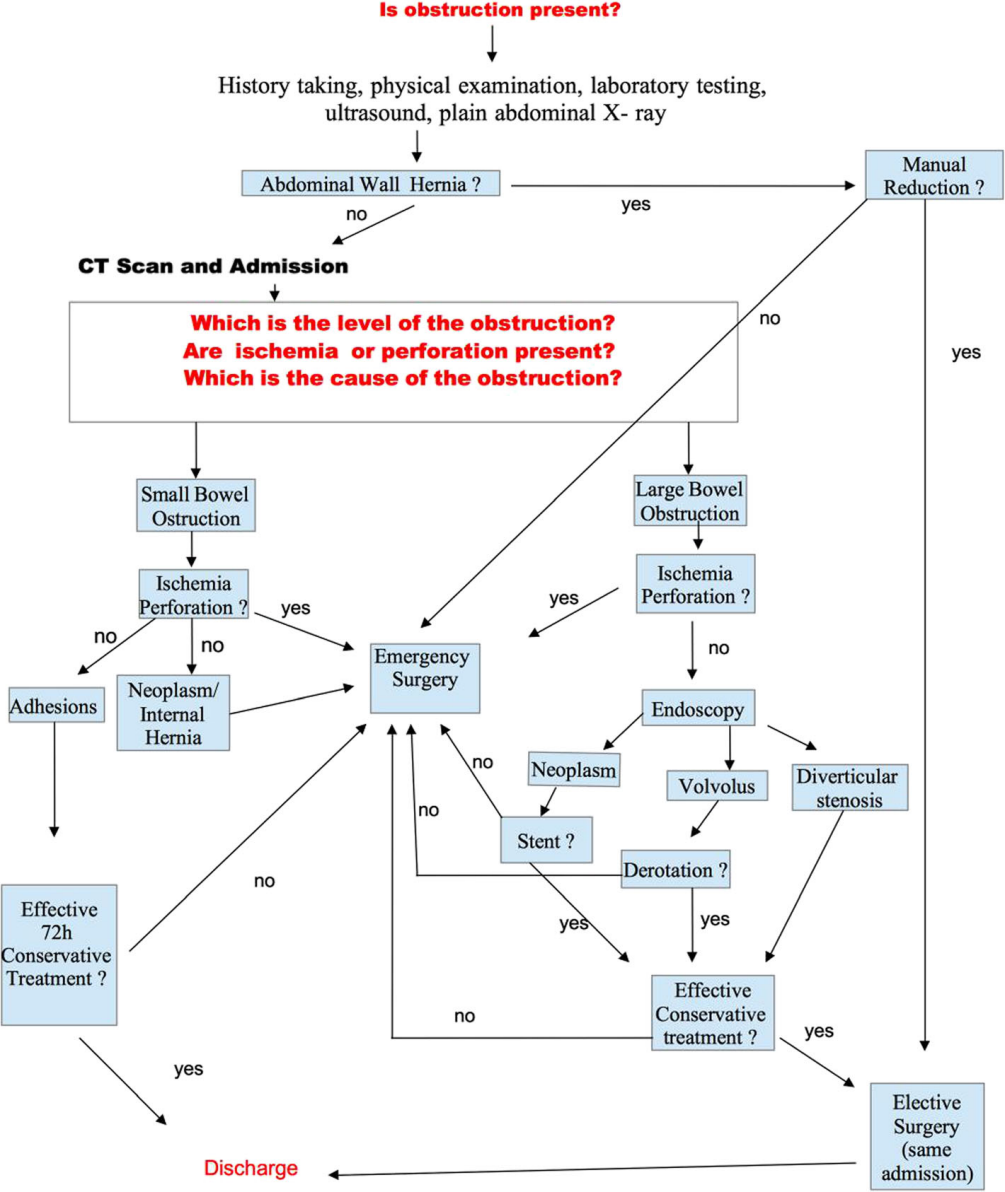
Management strategy of bowel obstruction (for about 90% of causes)

Questions include the following: How should this patient be evaluated and treated? What is the working diagnosis? Options include stump appendicitis, right colon diverticulitis, pelvic inflammatory disease, bowel obstruction, gastroenteritis, right renal colic, right colon cancer, bowel ischemia, or inflammatory bowel disease (Fig. 1).

## Discussion: strategies and evidence

### Initial patient assessment

A complete history along with physical exam and laboratory tests should be performed upon presentation to the emergency unit. Patients should be asked about their last defecation/bowel gas passage. Having a history of previous abdominal surgery has 85% sensitivity and 78% specificity to predict adhesive small bowel obstruction [9]. Previous diverticulitis episodes or chronic constipation history (dolicho-sigmoid) may suggest diverticular stenosis and volvulus, respectively. Previous events of rectal bleeding and unexplained weight loss are suggestive of colorectal cancer. Coexisting cardiopulmonary, renal, or hepatic comorbidities also require caution because they are associated with an increased surgical risk and may influence management strategies. Medications that could affect peristalsis are important for differential diagnosis because they are associated with pseudo-obstruction and adynamic ileus.

In bowel obstruction, abdominal pain is classically a colic onset due to an increase in motility to overcome occlusion. This is later replaced by continuous pain attributable to reduced peristalsis and dilation. Pain can be intense and untreatable with analgesics in case of ischemia (small bowel/large bowel volvulus) or perforation.

Nausea and emesis are earlier and more represented in small bowel obstruction. An abdominal examination can detect a strong predictive sign such as abdominal distension (sudden onset for volvulus or progressive for colorectal cancer) with a positive likelihood ratio of 16.8 and negative likelihood ratio of 0.27 [10]; peritonism signs are conversely associated with ischemia and/or perforation.

Each hernia orifice (umbilical, inguinal, femoral) and all laparotomic/laparoscopic incision scars should be carefully examined. Digital rectal examination and rectoscopy can be useful in patients to detect blood or a rectal mass suggestive of colorectal malignancy.

Anamnesis and clinical examination can be very difficult in elderly or unconscious patients. In these patients is fundamental the evaluation of vital signs with a cardiopulmonary examination: severe bowel obstruction can cause hypovolemic shock and in case of perforation, septic shock. Abnormal vital signs or the general appearance of the patient including facial expression, skin color and temperature, and altered mental activity should alert the clinician that a patient may be in critical conditions. The most common signs of shock include tachycardia, tachypnea, cool extremities, mottled or cyanotic skin, slow capillary refill, and oliguria.

A complete blood count, renal function and electrolytes (to exclude pre-renal acute renal failure), and liver function tests are suggested as the first laboratory tests. Low serum bicarbonate levels, low arterial blood pH, high lactic acid level, marked leukocytosis, and hyperamylasemia may be useful in the diagnosis of intestinal ischemia. A coagulation profile should be also tested because of the potential need for emergency surgery.

### Initial management

Supportive treatment must begin as soon as possible with intravenous crystalloids, anti-emetics, and bowel rest.

Isotonic dextrose-saline crystalloid and balanced isotonic crystalloid replacement fluids containing supplemental potassium in an equivalent volume to the patient’s losses are recommended. Nasogastric suction can be diagnostically useful to analyze gastric contents (a feculent gastric aspirate is a characteristic of distal small bowel obstruction or large bowel obstruction). Nasogastric suction can be also therapeutically important to prevent aspiration pneumonia decompressing the proximal bowel [11]. A Foley catheter should be also inserted to monitor urine output.

### Diagnostic evaluation (Tables 2 and 3)

#### Abdominal plain X-ray

Abdominal plain X-ray is the first level radiologic study. In small bowel obstruction, plain abdominal radiographic findings are diagnostic in 50–60%, inconclusive in 20–30%, and misleading in 10–20% of patients [12, 13].

**Table 2:** Procedures for the evaluation and treatment of bowel obstruction

	Advantages	Disadvantages
Plain abdominal X-ray	Availability	No etiologic diagnosis
Computed tomography scan	Etiologic diagnosis	Ionizing radiations exposure
Endoscopy (large bowel obstruction only)	Endoscopic treatment	Perforation risk
Conservative treatment	Fast recovery	Failure, complications, recurrence
Surgery	Etiologic treatment	Complications, stoma risk, recurrence

In one study after radiography, the sensitivity of bowel obstruction was significantly higher than after clinical evaluation only: 74% versus 57%, respectively (*P* < 0.01). However, the positive predictive value did not differ significantly between clinical assessment only and with plain radiographs [14].

In a review of 140 cases of suspected large bowel obstruction, the abdominal X-ray had 84% sensitivity and 72% specificity [15].

#### Water-soluble contrast administration X-ray

A water-soluble contrast enema has 96% sensitivity and 98% specificity in diagnosing large bowel obstruction [15] but cannot distinguish different large bowel obstruction causes.

A small bowel follow-through with water-soluble contrast is widely used in patients for adhesive small bowel obstruction non-operative management. Several systematic reviews and meta-analyses have established the utility of water-soluble contrast agents in the diagnostic work-up of adhesive small bowel obstruction [16–18]. If the contrast has not reached the colon on an abdominal X-ray 24 h after administration, then this is highly indicative of non-operative management failure [19]. Multiple studies have shown that the use of water-soluble contrast agents accurately predicts the need for surgery with an active therapeutic role [16, 17, 20, 21].

The administration of water-soluble contrast agents in adhesive small bowel obstruction is safe in terms of morbidity and mortality, but adverse effects due to their use have been reported. Potential life-threatening complications are aspiration pneumonia and pulmonary edema. To avoid these complications, the contrast medium should be administered when the stomach has been adequately decompressed through a nasogastric tube. Another potential adverse effect is that water-soluble contrast agents, because of higher osmolarity, may further dehydrate a patient with small bowel obstruction, shifting fluids into bowel lumen; in some children and elderly adults, the loss of plasma fluid may be sufficient to cause a shock-like state [22].

The contrast medium may be administered on the dosage of 50–150 ml, either orally or via nasogastric tube, and can be given both at immediate admission and after an attempt of initial traditional conservative treatment of 48 h. The practice of giving water-soluble contrast at 48 h may reduce both the risk of aspiration pneumonia and of dehydration because the patient should have been adequately rehydrated. In this situation, the contrast material can be diluted with water [22, 23].

Rare anaphylactoid reactions following the use of oral contrast media have been reported [22].

Caution in their administration may be warranted in patients at high risk of gastropathy [24].

#### Ultrasound

Small bowel obstruction can be diagnosed with ultrasound if there are > 2.5-cm dilated loops of the bowel that are proximal to collapsed loops of bowel and if there is decreased or absent peristalsis activity [25]. Using ultrasound for small bowel obstruction diagnosis has 90% sensitivity and 96% specificity [9].

Visualization of large bowel obstruction with ultrasound is as good as computed tomography. Computed tomography is clearly superior to ultrasound in terms of the etiologic definition for both small bowel obstruction and large bowel obstruction [26, 27]. Ultrasound performs better than planar abdominal X-ray in large bowel obstruction [28].

#### Computed tomography scan

**Fig. 2:**
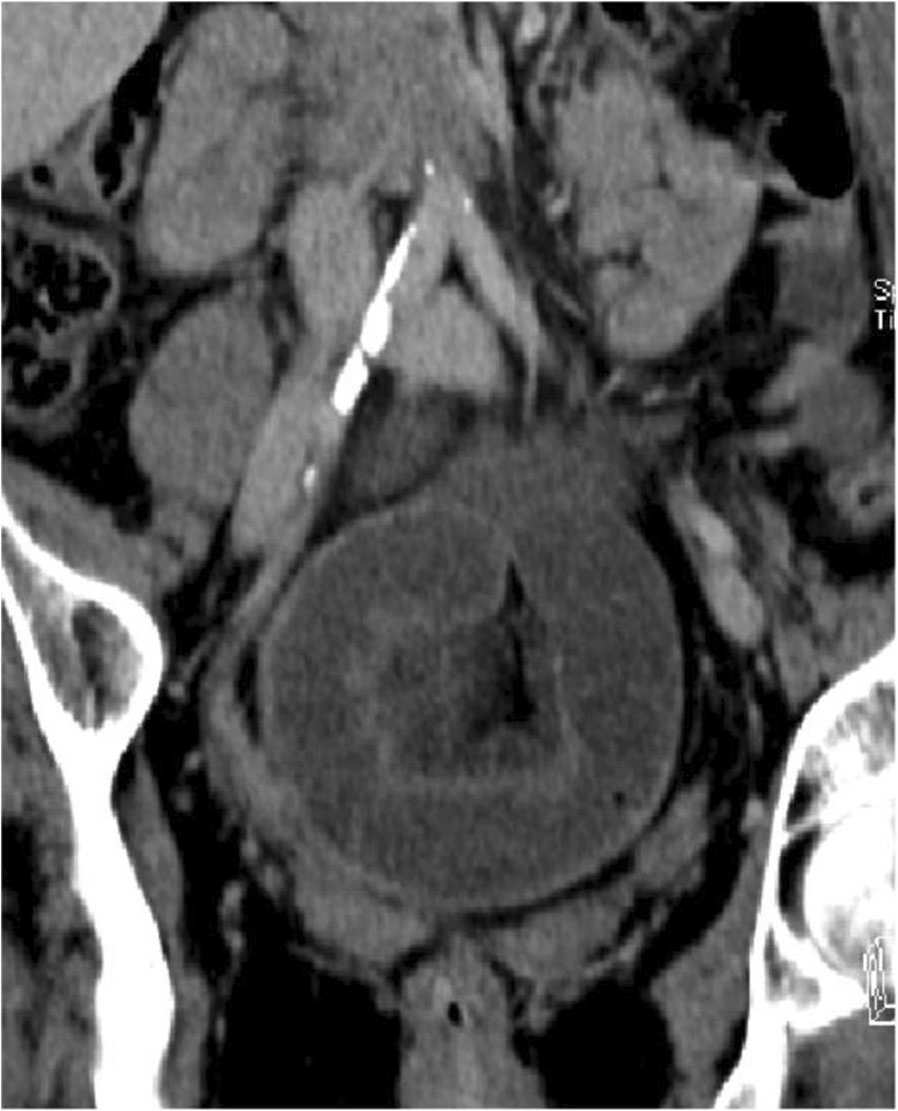
ASBO caused by single band adhesion: CT scan evidence

The diagnostic accuracy of computed tomography with intravenous contrast is superior to that of conventional abdominal radiography and ultrasound (Fig. 2). In addition to its higher sensitivity and specificity, an important advantage of computed tomography is its ability to provide information about the underlying cause of obstruction or to provide information about an alternative diagnosis if no signs of bowel obstruction are present. Computed tomography leads to more accurate management and assistance in preoperative planning [13].

Positive oral contrast material is not needed in the diagnosis of small bowel obstruction with computed tomography because the intraluminal fluid and gas already present within the obstructed bowel are excellent contrast agents. If positive oral contrast material has been given in patients with small bowel obstruction, then a delayed abdominal radiograph during non-operative management can assess if the contrast material has progressed to the colon.

When doubts about the large bowel obstruction diagnosis persist, a water-soluble rectal contrast agent can be administered to better visualize obstruction [29–31]. Computed tomography can also accurately stage neoplastic bowel obstruction and identify superimposed complications such as intestinal perforation and peritonitis [32].

In the case of bowel obstruction, it is critical to identify ischemia and necrosis—especially in adhesive small bowel obstruction and sigmoid volvulus. Computed tomography gives an excellent evaluation of the bowel wall, its vessels, and mesentery-mesocolon. Pneumatosis can identify coexistent ischemia and/or infarction.

The sensitivity varies between 75 and 100%, and specificities range from 61 to 93% [33].

The diagnosis of internal hernias is very difficult because of their nonspecific clinical picture [34]. Many different types of internal hernias have been described: paraduodenal, mesentery-related, greater omentum-related, lesser sac, mesocolon-related, pericecal, falciform ligament, pelvic internal, and Roux-en-Y anastomosis-related. An internal hernia can evolve into intestinal strangulation: accurate preoperative diagnosis is possible only with computed tomography [35].

#### Magnetic resonance imaging

To minimize the burden of ionizing radiation in children and pregnant women, magnetic resonance imaging is a valid alternative examination to computed tomography scan for bowel obstruction [36]: prospective study demonstrated a sensitivity of 95% and a specificity of 100% [37].

#### Colonoscopy

The role of colonoscopy is limited to the diagnosis of large bowel obstruction. The goal is to exclude other causes for obstruction. Biopsy should be performed in cases of suspected malignancy when emergency surgery has not been indicated or endoscopic stent placement can be expected [38]. In this case, carbon dioxide insufflation may be an acceptable alternative to conventional air insufflation to avoid prolonged abdominal bloating, excessive abdominal pain, and discomfort during colonoscopy. Besides, CO_2_ is absorbed from the colon 150 times faster than nitrogen and reduces the risk of the bowel ischemia resulting in reduced spasm and pain [39].

### Therapy

#### Conservative (non-surgical) therapy

Conservative treatment is the cornerstone of non-operative management in all patients with adhesive small bowel obstruction unless there are signs of intestinal ischemia/perforation. Evidence for the ideal duration of non-operative is lacking, but most authors consider a 72-h cutoff safe and appropriate [19]. The mainstay of non-operative management is nil per os and decompression with naso-gastric suction or long intestinal tube. There has been some discussion in the literature about the use in adhesive small bowel obstruction of long intestinal tubes: long trilumen naso-intestinal tubes are more effective than naso-gastric tubes, but they require endoscopic insertion [11].

Water-soluble contrast administration is a valid and safe treatment that correlates with a significant reduction in the need for surgery in patients with adhesive small bowel obstruction with also a significant reduction in the time to resolution and length of stay. The administration of water-soluble contrast is a secure treatment with no significant differences in complications or mortality [16, 17]. Adhesive small bowel obstruction recurrence is possible after non-operative management: 12% of non-operatively treated patients are readmitted within 1 year, and this value increases to 20% after 5 years [40].

In case of complicated hernia, a prompt manual reduction has to be attempted. Emergency surgery is needed for unsuccessful reduction [41, 42]. The same admission elective surgery is indicated for all patients submitted to successful manual reduction.

Diverticular obstruction follows multiple attacks of diverticulitis with marked fibrosis of the colon wall leading to narrowing and stricture formation; in some other cases, colonic obstruction can complicate acute diverticulitis due to edema narrowing. The site of obstruction is usually in the sigmoid colon; occlusion is normally incomplete and resolves with conservative treatment [43].

Sigmoid volvulus colonoscopy allows one to not only assess the viability of the sigmoid but to also achieve detorsion. If colonic necrosis is present, then the patient undergoes immediate surgery. In the absence of colonic necrosis, endoscopy can convert an urgent situation into an elective situation in the same admission. Colonoscopic detorsion is a simple and minimally invasive procedure with a success rate of 70 to 95% and a 4% morbidity. However, mortality is about 3% in a recent study with a recurrence rate of up to 71% [44, 45].

For palliation of obstructing left colon cancer, self-expanding metallic stents are preferred to colostomy because they are associated with similar mortality/morbidity rates but a shorter hospital stay [46]. Self-expanding metallic stents can be also a bridge to elective surgery for obstructing left colon cancer. It offers a better short-term outcome than emergency surgery because the rate of stomas is lower; long-term outcomes seem comparable, but there is still insufficient oncological evidence. Thus, self-expanding metallic stents should not be considered the treatment of choice for obstructing left colon cancer: it may be a valid option in selected cases and in centers with significant expertise [47–50].

#### Surgery

Prosthetic repair is the treatment of choice for most abdominal wall complicated hernias (inguinal, femoral, incisional, umbilical, epigastric, parastomal, spigelian, etc.).

In case of perforation/bowel resection with contaminated surgical fields, suture repair is preferred due to the risk of mesh infection. Diagnostic laparoscopy may be a useful tool to assess bowel viability after reduction of complicated hernias [51]. Repair of complicated hernia can be performed with a laparoscopic approach when no bowel resection anastomosis is needed, which normally requires a mini-open approach (small laparotomy) [51].

Internal hernias are treated with prompt reduction, suture repair, and bowel resection anastomosis in case of intestinal necrosis.

Historically, abdominal adhesiolysis through laparotomy has been the standard therapy for adhesive small bowel obstruction. In the case of emergent surgical exploration (i.e., perforation or bowel ischemia) or for conservative treatment failure, operative laparotomic surgery is the treatment of choice [6, 19, 52]. Laparoscopic adhesiolysis has been introduced in recent decades and can decrease morbidity in subgroups of patients undergoing surgery for adhesive small bowel obstruction. The risk of intestinal injuries is higher in laparoscopic surgery for adhesive small bowel obstruction. Therefore, careful selection of patients for laparoscopic surgery is mandatory. Results of randomized trials will soon be published [19, 53, 54]. The risk of recurrence is slightly lower after operative treatment compared to non-operative treatment: 8% after 1 year and 16% after 5 years [40, 55].

Small bowel obstruction caused by small bowel tumors (adenocarcinoma, neuroendocrine tumors, gastrointestinal stromal tumors, and lymphomas) is treated with resection and anastomosis. Oncologic management of these tumors must be obviously considered following the same schemes of tumors that arise outside the small bowel [56–58].

For large bowel obstruction caused by sigmoid volvulus without ischemia or perforation, the best strategy is an endoscopic detorsion procedure followed by same admission surgery that includes a sigmoid colectomy with primary anastomosis. Exclusively endoscopic therapy without subsequent surgery must be reserved for high-surgical-risk patients. In case of ischemic volvulus or failed derotation, surgery has to be performed as soon as possible. In cecal volvulus, endoscopy has no role, and surgery (right hemicolectomy) is the only option [44]. The role of laparoscopic surgery for volvulus is limited: the absence of fixation of the sigmoid colon and its excessive length often make laparoscopic exposure and dissection difficult.

Resection and primary anastomosis are the desired procedure for diverticular large bowel obstruction, and it should be attempted regardless of bowel preparation after a successful conservative treatment in the same admission [59]. Exclusively conservative therapy or Hartmann procedure could be more appropriate for high-risk patients.

Resection and primary anastomosis are the best options for malignant large bowel obstruction in the absence of significant risk factors or perforations. Patients with high surgical risk or perforations are better managed with staged procedure (e.g., Hartmann procedure). Many prospective and retrospective studies into resection and primary anastomosis in malignant large bowel obstruction have reported percentages of anastomotic leaks ranging from 2.2 to 12% [60, 61] comparable to the 2–8% rate after elective surgical procedures. In the case of large bowel obstruction caused by extraperitoneal rectal cancer, resection of the primary tumor should be postponed and a stoma should be fashioned to permit a correct staging and a more appropriate oncological neoadjuvant treatment. Laparoscopy in emergency treatment of malignant large bowel obstruction should be reserved to selected cases in specialized centers [62–64].

### Other uncommon bowel obstructions

Carcinomatosis, endometriosis, inflammatory bowel disease stenosis, intussusception, post-ischemic stenosis, radiation stenosis, postanastomotic stenosis, gallstones, foreign bodies, bezoars, and tuberculosis can cause bowel obstruction in a minority of cases (globally 10–15%). Here, a computed tomography scan is critical for diagnosis. Conservative treatment should be started in the absence of ischemia or perforation, but surgery is needed as a rescue therapy for failed conservative treatments or as elective therapy to prevent recurrence [65].

### Areas of uncertainty

Bowel obstruction is a common and challenging surgical emergency. Further studies are needed to evaluate more precisely the role of conservative treatment in adhesive small bowel obstruction and its length. Moreover, a great debate is underway about laparoscopic adhesiolysis potentialities against possible severe technical complications. Both issues are critical to decrease the well-known adhesive small bowel obstruction recurrence risk. Stents will probably become the mainstay of cancer large bowel obstruction transforming emergency operations in elective cases decreasing complications and stomas.

### Guidelines

The recommendations in this article are concordant with guidelines published by the World Society of Emergency Surgery [19] (Table 3).

**Table 3:** Key clinical points

- *Multidetector computed tomography* has emerged as the best imaging test for the diagnosis of mechanical bowel obstruction and its complications and can help patients’ management to either conservative or operative management - *Conservative adhesive small bowel obstruction treatment* is the mainstay non-operative management in all patients with adhesive small bowel obstruction without signs of perforations or bowel ischemia - *Self-expanding metallic stents as bridge to elective surgery* for obstructing left colon cancer offers a better short-term outcome than direct emergency surgery because morbidity is comparable, but rate of stomas is significantly lower; long-term outcomes seem also comparable, but there is still insufficient oncologic scientific evidence to prove it - *Colonoscopic sigmoid volvulus detorsion* is a simple and mini-invasive procedure associated with a success rate of 70 to 95% with a 4% morbidity: it can convert an urgent situation into an elective one but with high recurrence rate.	

## Conclusions

Bowel obstruction is the most likely diagnosis in the patient described in the vignette. High white blood cells and peritonism could suggest adhesive small bowel obstruction with possible ischemia. A computed tomography scan with intravenous contrast must be performed as soon as possible. If computed tomography scan confirms adhesive small bowel obstruction with ischemia or perforation, then the patient must go to surgery as soon as possible: a laparoscopic approach should be attempted.

If computed tomography shows adhesive small bowel obstruction without ischemia or perforation, then a conservative treatment should be initiated: nasogastric suction and fluid replacement therapy have to be performed with a scrupulous wait-and-see strategy. After gastric contents are cleared, a water-soluble contrast administration challenge should be performed. The patient has to be monitored with regard to ischemia (peritonism, white blood cells, lactate). Surgery has to be performed immediately in the case of clinical deterioration—preferably beginning with a laparoscopic technique.

After 24 h, a plain abdominal X-ray should be performed to determine if oral contrast reached the large bowel: if positive, then oral nutrition can be started. If negative, non-operative management could be continued for another 48 h: surgery should be performed after this limit—preferably starting with a laparoscopic approach.

## Abbreviations

ASBO: Adhesive small bowel obstruction
CT: Computed tomography
SBO: Small bowel obstructions
